# ICD‐11 for Alcohol Use Disorders: Not a Convincing Answer to the Challenges

**DOI:** 10.1111/acer.14182

**Published:** 2019-09-05

**Authors:** Jürgen Rehm, Markus Heilig, Antoni Gual

**Affiliations:** ^1^ Institute for Mental Health Policy Research Centre for Addiction and Mental Health (CAMH) Toronto Ontario Canada; ^2^ Campbell Family Mental Health Research Institute CAMH Toronto Ontario Canada; ^3^ Dalla Lana School of Public Health University of Toronto Toronto Ontario Canada; ^4^ Department of Psychiatry University of Toronto Toronto Ontario Canada; ^5^ Institute of Medical Science University of Toronto Toronto Ontario Canada; ^6^ Institute of Clinical Psychology and Psychotherapy Center for Clinical Epidemiology and Longitudinal Studies Technische Universität Dresden Dresden Germany; ^7^ Department of International Health Projects Institute for Leadership and Health Management I. M. Sechenov First Moscow State Medical University Moscow Russian Federation; ^8^ Center for Social and Affective Neuroscience Department of Clinical and Experimental Medicine Linköping University Linköping Sweden; ^9^ Addictions Unit Psychiatry Department Neurosciences Institute, Hospital Clínic Barcelona Spain; ^10^ IDIBAPS (Institut per a la Recerca Biomèdica Agustí Pi i Sunyer) Barcelona Spain; ^11^ Red de Trastornos Adictivos Instituto Carlos III Madrid Spain

The latest revision of the International Classification of Diseases (ICD‐11) was published June 18, 2019 and includes a revised definition for alcohol use disorders (AUDs) and, more specifically, for alcohol dependence and the “harmful patterns of alcohol use.” In a recent issue, Saunders and colleagues (2019) reviewed the process employed in deciding upon these revisions and embedded it into an historical context. While the authors have not stressed this point, it should be clear that “all AUD diagnostic systems are fallible simplifications of complex and not completely understood phenomena that were developed for clinicians to use and not for researchers” (Schuckit, 2019, personal communication; see also World Health Organization, 1957, Room, 1998).

We will examine the revised ICD‐11 with respect to AUDs in light of the criticisms leveled at earlier versions of the ICD, including the following questions:

- Did the new criteria include underlying brain processes (Kwako et al., 2016, 2019)?
- Was the criticism regarding nonspecific consequences remedied in the revision (Martin et al., 2014)?
- Were the criteria changed to address issues of cultural specificity (Rehm and Room, 2017)?
- Is the level of alcohol use adequately incorporated in the revision (Lachenmeier et al., 2013)?
- Have any attempts been made to close the gap between the 2 major classification systems (Carvalho et al., 2019)?
- Is it likely that the ICD‐11 and its criteria will actually be used in clinical practice in primary care, where most patients with patterns of hazardous or harmful use or AUDs first appear within the treatment system?

## Did the New Criteria Include Underlying Brain Processes?

AUDs, like other diagnoses in mental health, are currently based mostly on behavioral symptoms, although there has been a push to change this and identify functional domains which link to brain processes (Hyman, 2007). As such, the DSM‐5 working groups were instructed to base criteria on neurobiological processes where possible.

For AUDs and other addictive disorders, Kwako and colleagues (2016) developed a neuroscience‐based framework and identified 3 neurofunctional domains—cognitive control (executive function), incentive salience, and negative emotionality—which form the core elements of AUDs. There is some empirical support for mapping these domains onto the current diagnostic criteria (Kwako et al., 2016, 2019), but much work still needs to be done to determine whether they are sufficiently sensitive and specific to serve as diagnostic criteria by themselves. Whether the use of these domains adequately captures the heterogeneity of what we currently call AUDs is an empirical question that will require large field trials (Clark et al., 2017). Similarly, empirical data will be needed to determine the ability of these functional domains to distinguish between AUDs and other disorders.

In addition, 1 dimension present both in the DSM and in the ICD in its current iteration—the social dimension—seems to be lacking. There is an emerging realization in neuroscience that the pathology of social cognition and function is a core element of addictive disorders, and will need to be linked to neural function (Heilig et al., 2016). Recent attempts at modeling these processes in laboratory animals have been successful. To date, however, these strategies have not linked complex addiction‐related social behaviors to their molecular and neural circuit substrates to a degree that would allow them to form the basis of clinical diagnostic criteria.

In summary, it may have been worthwhile for the ICD‐11 to at least attempt to capture a single‐core functional element in its definition or measurement, but, clearly, a full definition of AUDs based on neuroscience presently remains as elusive as it is appealing.

## Was the Criticism Regarding Nonspecific Consequences Remedied in the Revision?

Martin and colleagues (2014) criticized the use of consequences as criteria for substance use disorders (such as a failure to fulfill role obligations; reduced social activities in favor of substance use; continued use despite knowledge of social/interpersonal problems; and continued use despite knowledge of physical/psychological problems). They argue that these consequences are determined by context, and thus, there can be no universal criteria; they are determined via multiple causes and are thus nonspecific; and for some there is a lack of evidence that they are caused or exacerbated by substance use. While their criticisms are mainly exemplified by the DSM‐5, they also apply to the ICD‐11, in its criterion of “giving up other activities and pleasures” (Saunders et al., 2019).

## Were the Criteria Changed to Address Issues of Cultural Specificity?

Any kind of behavioral criteria risks being culturally specific. For the ICD‐11, this is an issue as 1 of the 3 criteria for an AUD diagnosis centers around “impaired control” (Saunders et al., 2019). Impaired control has different connotations in different cultures. While it clearly has negative connotations and it is almost taboo to admit to impaired control in some cultures, such as in Italy or other Mediterranean countries, in yet other cultures with patterns of occasional heavy drinking, it is actually one of the reasons to drink alcohol. In the latter cultures, people will agree to items of impaired control more easily, contributing to prevalence differences in alcohol dependence, which are more than 10‐fold between countries such as Italy and Latvia, despite the fact that the adult exposure of alcohol is less than twice as high, and the liver cirrhosis rates are almost 3 times higher in the latter (Rehm et al., 2013, 2015b).

While these are ecologic data, they seem to indicate that AUDs are impacted by operational definitions unrelated to the underlying diagnostic criteria. Overall, the only moderate correlation between adult *per capita* consumption and AUDs—with an explained variance of less than 40%—also corroborates this conclusion (the 40% value was derived from a Pearson correlation of values for all countries, based on Manthey et al., 2019). At least for epidemiologic research, it is probably wiser to rely on heavy drinking levels over time to determine the prevalence of AUDs rather than depending on surveys which ask for behavioral criteria in a stepwise fashion, and then construct a “diagnosis” based on the responses (Rehm, 2016).

## Was the Level of Alcohol Use Adequately Incorporated in the Revision?

The level of alcohol use is the main component of variance in the WHO Alcohol Use Disorders Identification Test (Bush et al., 1998), the main variable used by clinicians to detect hazardous drinking or AUDs (Rehm et al., 2015a), the main criterion used to test the effectiveness of treatment accepted by major regulators (European Medicines Agency, 2010; Hasin et al., 2017), and a major determinant of harm (Nutt and Rehm, 2014). Yet, the level of alcohol use is missing from *any* criteria for AUDs in either the ICD or the DSM diagnostic systems. This situation has triggered a call to redefine AUDs based on heavy drinking levels over time, as all the other behavioral and neurobiological symptoms are consequences of it (Rehm et al., 2013, 2014).

In the DSM‐5, the exclusion of level of consumption was briefly discussed (Hasin et al., 2013). The authors of the main rationale for final criteria in the DSM‐5 concluded that although the inclusion of a consumption item was shown to fit well into a 1‐dimensional model for alcohol (Saha et al., 2007, see also Borges et al., 2010), it had different item severities cross‐culturally (e.g., Shmulewitz et al., 2010). However, this does not seem to be a valid argument: First, the criteria for AUDs in the DSM‐5 do not constitute a Rasch scale (Hasin et al., 2013) of items (criteria) with noncrossing information characteristic curves, and second, in the large WHO international validation study of substance use diagnostic systems and instruments, the severity of most items consistently changed between cultures (Schmidt and Room, 1999). In any case, the number of criteria met in DSM diagnoses and the level of drinking showed almost perfect correlations (Rehm et al., 2014).

While we cannot repeat the arguments and discussions about inclusion of level of drinking exhaustively here, it should suffice to note that level of drinking over time is probably one of the best indicators of AUDs, and should be included in the diagnoses of AUDs as it best determines both the course of illness and the subsequent burden including mortality.

## Were any Attempts Made to Close the Gap Between the 2 Major Classification Systems?

AUDs were defined in a relatively similar manner in the DSM‐IV (American Psychiatric Association, 1994) and the ICD‐10 (World Health Organization, 1992), mainly with respect to the diagnosis of alcohol dependence (Clark et al., 2017; Üstün et al., 1997). However, within the DSM‐5 (American Psychiatric Association, 2013) and the ICD‐11 (Saunders et al., 2019), the gap between the 2 systems widened once again, with 1 system describing AUDs as a continuous disorder with a low threshold and a combination of relatively unspecific behavioral symptoms of dependence and abuse, and the other reiterating a categorical approach highlighting key criteria.

While we prefer the ICD‐11 approach because of the DSM‐5's multitude of nonspecific behavioral items combined with a low consumption threshold, the gap between these 2 diagnostic systems is problematic and concerns especially—but not exclusively—the lack of convergence between the ICD‐11's diagnosis of “patterns of harmful drinking” and the DSM‐5 criteria (e.g., Chung et al., 2017; Degenhardt et al., 2019; Lago et al., 2016).

This gap does not help health professionals in designing interventions for AUDs (Room and Rehm, 2019), although it seems that health professionals do use different criteria altogether in their clinical work (see below and Ref. Rehm et al., 2015a). In summary, despite numerous appeals for greater alignment, and assurances that the 2 systems do in fact converge or are essentially the same, the reality is different: With the DSM‐5 and the ICD‐11, we are now 1 step further in widening the gap between the diagnostic systems.

## Is it Likely That the ICD‐11 and its Criteria Will Actually be Used in Clinical Practice in Primary Care, Where Most of the Patients With Patterns of Hazardous or Harmful Use First Appear Within the Treatment System?

Primary care could play a major role in the treatment of AUDs, not only for detection, provision of brief advice for patients exhibiting symptoms of hazardous use, and referral, but also as the place where most AUDs should be treated (Rehm et al., 2016; Carvalho et al., 2019). However, primary healthcare physicians and other professionals are not guided by current diagnostic systems, but rather by patients reporting heavy consumption over time and telltale physical signs or comorbidities (Rehm et al., 2015a). In fact, in a large European study carried out in 8 countries, there was only limited overlap between formal and physician diagnoses, and only 30% of diagnoses of alcohol dependence made by a formal instrument were also detected by physicians. However, the reverse was also true: Only about 40% of the people detected by primary healthcare physicians based on criteria such as consumption level, smell of alcohol on the patient's breath, the presence of red eyes, and findings of elevated liver enzymes or other comorbidities would qualify for a diagnosis with formal instruments. Modern biomarkers such as phosphatidylethanol PEth could be used to assess the level of drinking in the event that a clinician doubted the validity of a patient's self‐report (Carvalho et al., 2019). However, it is unlikely that ICD‐11 will effect such a change in clinical practice, as it is based on very similar criteria as the ICD‐10.

The inclusion of dependence in the ICD‐11 may be of help in defining AUDs in specialized care when compared to the DSM‐5. However, this concerns only the most severe cases of AUDs and is not in line with the statement that the guiding principle for the development of the ICD‐11 was for improvements in public health (i.e., the new diagnostic system would help establish an effective treatment system to minimize the overall burden of AUDs). Since the ICD‐11 explicitly aimed at contributing to public health in the area of AUDs (Poznyak et al., 2018), it must be evaluated by these standards.

The establishment of a single diagnosis for AUDs with high thresholds for consumption may in fact hurt public health by perpetuating or possibly even increasing stigma (i.e., “normalized drinking” of the majority vs. those who suffer dependence), which is already among the highest for all mental disorders (Schomerus et al., 2014). The concept of dimensionality and associated continuum beliefs have been shown to reduce stigma (Schomerus et al., 2013).

The other diagnoses in the ICD‐11 system also do not provide help here. A pattern of harmful drinking is defined as “a pattern of alcohol use that has caused damage to a person's physical or mental health or has resulted in behaviour leading to harm to the health of others.” (Saunders et al., 2019). ICD‐11 then goes on to define direct or secondary toxic effects on body organs and systems as one of the manifestations of harm. The problem here is that *any* use of alcohol *can*—but not necessarily does—cause damage to physical health (Rehm et al., 2017), but a causal relationship between the 2 at the individual level is not that easy to determine.

As an example, one can consider the relationship between alcohol and the risk of hypertension where 1 drink per day on average has been shown to increase blood pressure and the risk of hypertension (Roerecke et al., 2018). Consider a patient with hypertension and alcohol consumption: There is a good chance that her or his hypertension has been caused or worsened by alcohol consumption, fulfilling the definition of “harmful drinking.” But is such a diagnosis clinically meaningful when we cannot draw a definitive link?

For probabilistic relationships such as the one between alcohol use and hypertension, population relationships can readily be assessed, but it is not as clear for an individual patient. The same is true for seemingly more clear‐cut cases such as the relationship between heavy alcohol use and liver cirrhosis, where it is very likely—but again not absolutely clear—whether the alcohol consumption caused the liver condition. Accordingly, the definition of harmful drinking is problematic in that it cannot be applied with certainty.

Thus, from a public health point of view, it might make more sense to go into a continuous and probabilistic framework of health harms of alcohol use based on the level of drinking linked to absolute and relative risks. Such a concept would also make sense in the clinical context of screening of patients. If screening is based on the level of alcohol use, which is a continuous and fluctuating variable, it could help us define patterns of harmful use more clearly in terms of disease consequences and public health.

## Conclusion

In summary, while the ICD‐11 is of help in defining the most severe end of the AUD spectrum, it will need to be complemented with other instruments in order to improve future prevention and public health measures, and both of these goals were explicitly expected to be part of this revision (Poznyak et al., 2018; Saunders et al., 2019). The ICD‐11 also does not answer some of the most pressing questions posed in the current literature on AUDs. Overall, the self‐imposed challenge of trying to make a classification of AUDs, which is clinically used and public health‐relevant, does not seem to have been accomplished with this edition. In our view, the easiest way to have improved on this would simply have been to include the level of alcohol use as part of the AUD diagnostic criteria.

## Conflicts of Interest

JR reports grants from Lundbeck pharmaceutical company, and grants from D & A pharmaceutical company, outside the submitted work. MH reports serving on advisory boards for Indivior, Aelis Pharma, Adial Pharmaceuticals, and BrainSway Technology. AG reports grants from Lundbeck pharmaceutical company, grants from D & A pharmaceutical company, and grants from Novartis pharmaceutical company, outside the submitted work.
